# Crystal structure and Hirshfeld surface analysis of 2-(2-oxo-3-phenyl-1,2,3,8a-tetra­hydro­quinoxalin-1-yl)ethyl acetate

**DOI:** 10.1107/S2056989021005247

**Published:** 2021-05-21

**Authors:** Nadeem Abad, Lhoussaine El Ghayati, Camille Kalonji Mubengayi, El Mokhtar Essassi, Savaş Kaya, Joel T. Mague, Youssef Ramli

**Affiliations:** a Laboratory of Medicinal Chemistry, Drug Sciences Research Center, Faculty of Medicine and Pharmacy, Mohammed V University in Rabat, Morocco; b Department of Biochemistry, Faculty of Education & Science, Al-Baydha University, Yemen; c Laboratoire de Chimie Organique Heterocyclique, Faculté des Sciences, Université Mohammed V in Rabat, Morocco; d Laboratoire de Chimie et Biochimie de l’Institut Superieur des Techniques Medicales de Kinshasa, Republique Democratique du , Congo; e Sivas Cumhuriyet University, Health Services Vocational School, Department of Pharmacy, 58140, Sivas, Turkey; f Department of Chemistry, Tulane University, New Orleans, LA 70118, USA

**Keywords:** crystal structure, di­hydro­quinoxaline, hydrogen bond, π-stacking, C—H⋯π(ring)

## Abstract

In the title mol­ecule, C_18_H_16_N_2_O_3_, the di­hydro­quinoxaline moiety, with the exception of the N atom is essentially planar with the inner part of the methyl­propano­ate group (CH_2_—CH_2_—O) nearly perpendicular to it. In the crystal, inversion dimers formed by C—H⋯O hydrogen bonds are connected into oblique stacks by π-stacking and C—H⋯π(ring) inter­actions.

## Abstract 2

The di­hydro­quinoxaline moiety, with the exception of the N atom, is essentially planar with the attached phenyl ring inclined to it by 11.64 (6)° and the inner part of the methyl­propano­ate group nearly perpendicular to it. In the crystal, inversion dimers formed by C—H⋯O hydrogen bonds are connected into oblique stacks by π-stacking and C—H⋯π(ring) inter­actions.

## Chemical context   

**Figure 1:**
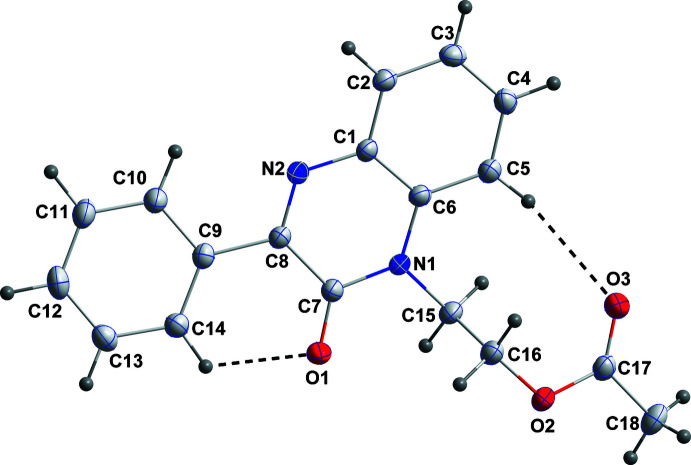
The title mol­ecule with labelling scheme and 50% probability ellipsoids. The intra­molecular C—H⋯O hydrogen bonds are shown by dashed lines.

Quinoxaline are a class of nitro­gen containing heterocyclic compounds, found in many biologically active drugs (Ramli & Essassi, 2015 ▸; Ramli *et al.*, 2014 ▸). In addition, this heterocyclic scaffold possess anti­corrosion characteristics (El Ouali *et al.*, 2010 ▸; Zarrok *et al.*, 2012 ▸; Tazouti *et al.*, 2016 ▸; El Aoufir *et al.*, 2016 ▸; Laabaissi *et al.*, 2019 ▸). In a continuation of our recent work focused on the synthesis and biological evaluation of novel heterocyclic compounds (Guerrab *et al.* 2019 ▸, 2020 ▸, 2021 ▸; Abad *et al.*, 2021*a* ▸,*b* ▸; Missioui *et al.* 2021 ▸) we report here the crystal structure of the title compound (Fig. 1 ▸). As with many biologically active mol­ecules, the mol­ecular conformation adopted may have a significant effect on its activity.
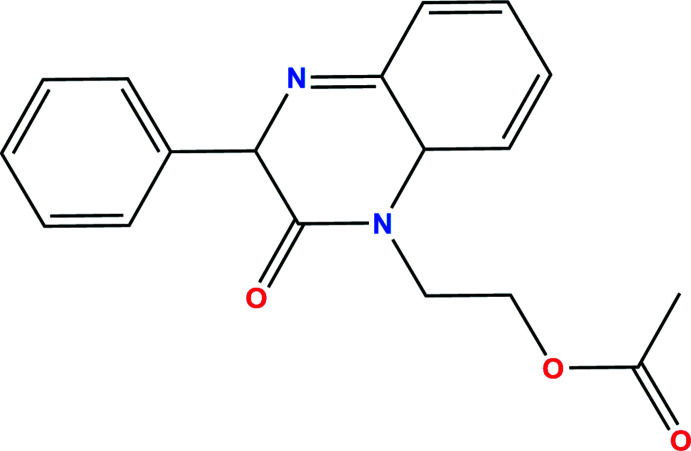


## Structural commentary   

The di­hydro­quinoxaline moiety, with the exception of N1, is planar to within 0.0186 (9) Å (r.m.s. deviation of the nine fitted atoms = 0.0116 Å). N1 lies 0.0526 (12) Å below the mean plane. The C9–C14 phenyl ring is inclined to the above plane by 11.64 (6)° while the inner part (CH_2_—CH_2_—O) of the methyl propano­ate substituent is nearly perpendicular to the di­hydro­quinoxaline unit, as indicated by the angle of 87.34 (6)° between the N1/C15/C16/O2 and N2/C1–C8 planes. The overall conformation is determined in part by the intra­molecular C5—H5⋯O3 and C14—H14⋯O1 hydrogen bonds (Table 1 ▸ and Fig. 1 ▸).

## Supra­molecular features   

**Figure 2:**
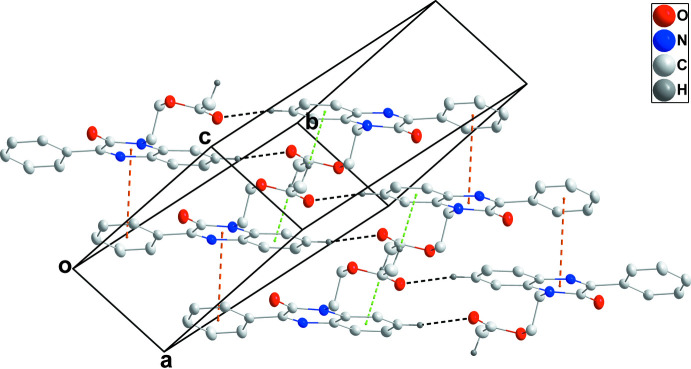
Perspective view of the packing. Inter­molecular C—H⋯O hydrogen bonds are shown by black dashed lines while π-stacking and C—H⋯π(ring) inter­actions are shown, respectively, by orange and green dashed lines.

In the crystal, inversion dimers are formed by C4—H4⋯O3^i^ hydrogen bonds [Table 1 ▸; symmetry code: (i) −*x* + 2, −*y* + 1, −*z* + 1] and are connected into oblique stacks by a combination of π-stacking inter­actions between the C1/C6/N1/C7/C8/N2 and C9–C14 rings [centroid–centroid distance = 3.7786 (9) Å, dihedral angle = 12.20 (6)°] and in addition C16—H16*B*⋯O3^ii^ and C18—H18*A*⋯*Cg*2^iii^ inter­actions [Table 1 ▸ and Fig. 2 ▸; *Cg*2 is the centroid of the C1–C6 ring; symmetry codes: (ii) *x* − 1, *y*, *z*, (iii) −*x* + 1, −*y* + 1, −*z* + 1]. The crystal packing also shows a C17=O3⋯*Cg*2 inter­action [O3⋯*Cg*2 = 3.9578 (12) Å, C17⋯*Cg*2 = 3.7440 (16) Å, C17=O3⋯*Cg*2 = 71.04 (8)°].

## Database survey   

A survey of the Cambridge Structural Database (Version 5.42, last update February 2021; Groom *et al.*, 2016 ▸) using the search fragment **II** yielded 30 hits of which those most similar to the title mol­ecule have the formula **III** with *R* = Me and *R*′ = CH_2_CO_2_H (DEZJAW; Missioui *et al.*, 2018 ▸), CH_2_C≡CH (DUCYUW; Benzeid *et al.*, 2009*a* ▸), benzyl [DUSHUV (Ramli *et al.*, 2010*b* ▸) and DUSHUV01 (Ramli *et al.*, 2018 ▸)], Et (IGANOU; Benzeid *et al.*, 2008 ▸), CH_2_CH=CH_2_ (YUPXAJ; Ramli *et al.*, 2010*a* ▸), with *R* = CF_3_ and *R*′ = *i*-Bu (DUBPUO; Wei *et al.*, 2019 ▸), with *R* = Ph and *R*′ = CH_2_(*cyclo*-CHCH_2_O) (NIBXEE; Abad *et al.*, 2018*a* ▸), benzyl (PUGGII; Benzeid *et al.*, 2009*b* ▸), CH_2_CH_2_CH_2_OH (RIRBOM; Abad *et al.*, 2018*b* ▸), CH_2_CO_2_Et (XEXWIJ; Abad *et al.*, 2018*c* ▸), CH_2_CH=CH_2_ (YAJGEX; Benzeid *et al.*, 2011 ▸) and with *R* = 3-NO_2_-C_6_H_4_ and *R*′ = benzyl (XIKHAD; Das *et al.*, 2018 ▸).
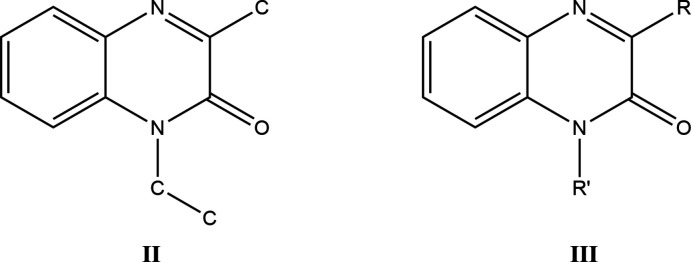


In the majority of the hits, the di­hydro­quinoxaline ring is essentially planar with the dihedral angle between the constituent rings being less than 1° or having the nitro­gen bearing the exocyclic substituent less than 0.03 Å from the mean plane of the remaining nine atoms. Two notable exceptions are DEZJAW, where the dihedral angle between the two rings is 3.32°, and RIRBOM, where the nitro­gen bearing the exocyclic substituent deviates by 0.062 Å from the plane defined by the other nine atoms.

## Hirshfeld surface analysis   

**Figure 3:**
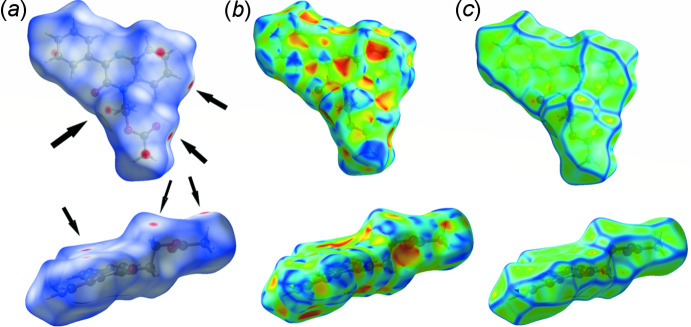
Front (top) and side (bottom) views of the Hirshfeld surface plotted over (*a*) *d*_norm_, (*b*) shape-index and (*c*) curvature.

**Figure 4:**
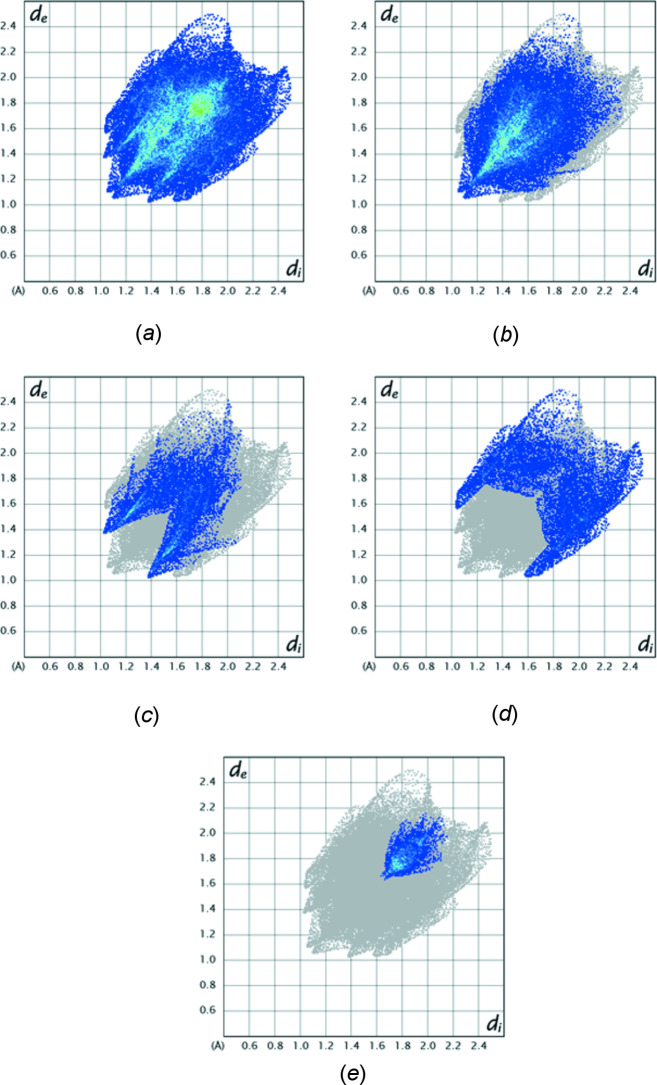
Two dimensional fingerprint plots showing (*a*) all inter­molecular inter­actions and those delineated into (*b*) H⋯H, (*c*) H⋯O/O⋯H, (*d*) H⋯C/C⋯H and (*e*) C⋯C inter­actions.

An effective means of probing inter­molecular inter­actions is Hirshfeld surface analysis (McKinnon *et al.*, 2007 ▸; Spackman & Jayatilaka, 2009 ▸), which can be conveniently carried out with *Crystal Explorer 17* (Turner *et al.*, 2017 ▸). A detailed description of the use of *Crystal Explorer 17* and the plots obtained has been published (Tan *et al.*, 2019 ▸) and will not be given here. Fig. 3 ▸ *a* presents front (top) and side (bottom) views of the Hirshfeld surface plotted over *d*_norm_ in the range −0.1367 to 1.2965 a.u. One of the intra­molecular C—H⋯O hydrogen bonds is indicated by the arrow at the left in the front view while those leading to the formation of the inversion dimers are shown by the arrows on the right of the front view. The C—H⋯π(ring) inter­action and the π-stacking inter­actions are represented by the red spots designated by arrows in the side view. Fig. 3 ▸ *b* presents the same two views of the surface plotted over the shape-index. In the front view, the π-stacking inter­action is evident at the center as an orange triangle surrounded by blue triangles. Fig. 3 ▸ *c* has the same two views of the surface plotted over the curvature index, with the flat area in the center indicating the locus of the π-stacking inter­action. Fig. 4 ▸ presents fingerprint plots for all inter­molecular inter­actions (*a*) and those delineated into H⋯H contacts (*b*, 49.4%), H⋯O/O⋯H contacts (*c*, 18.2%), H⋯C/C⋯H contacts (*d*, 17.8%) and C⋯C contacts (*e*, 7.2%).

## Synthesis and crystallization   

To a solution of 2-oxo-3-phenyl-1,2-di­hydro­quinoxaline (0.5 g, 2.25 mmol) in *N*,*N*-di­methyl­formamide (15 ml) were added 2-bromo­ethyl acetate (0.4 ml, 2.25 mmol), potassium carbonate (0.31 g, 2.25 mmol) and a catalytic qu­antity of tetra-*n*-butyl­ammonium bromide. The reaction mixture was stirred at room temperature for 24 h. The solution was filtered and the solvent removed under reduced pressure. The residue thus obtained was chromatographed on a silica gel column using a hexa­ne/ethyl acetate 9.5: 0.5 mixture as eluent. The solid obtained was recrystallized from ethanol solution to afford colorless column-like specimen of the title compound. Yield: 0.50 g, 67%; m.p. 471–473 K.

^1^H NMR (Bruker Avance 300 MHz, CDCl_3_) δ (ppm): 8.24 (*d*, 2H, Ar—H); 7.91 (*d*, 1H, Ar—H); 7.82 (*m*, 3H, Ar—H); 7.53 (*m*, 1H, Ar—H); 7.25 (*m*, 2H, Ar—H); 4.73 (*t*, 2H, O—CH_2_); 3.92 (*t*, 2H, N—CH_2_); 2.23 (*s*, 3H, OCOCH_3_).

^13^C NMR (Bruker Avance 75 MHz, CDCl_3_) δ (ppm):46.15 (N—CH_2_); 61.15 (O—CH_2_); 114.38, 123.82, 127.01, 127.72, 128.13, 128.96, 129.68, 130.33, 130.45,130.62 (CH—Ar); 132.78, 133.36, 135.40, 136.05, 154.24(Cq); 156.92 (C=O); 177.82 (O—C=O).

## Refinement   

Crystal data, data collection and structure refinement details are summarized in Table 2 ▸. All hydrogen atoms were located from a difference electron-density map and freely refined.

## Supplementary Material

Crystal structure: contains datablock(s) global, I. DOI: 10.1107/S2056989021005247/vm2249sup1.cif

Structure factors: contains datablock(s) I. DOI: 10.1107/S2056989021005247/vm2249Isup2.hkl

Supporting information file. DOI: 10.1107/S2056989021005247/vm2249Isup3.cml

CCDC reference: 2062956

Additional supporting information:  crystallographic information; 3D view; checkCIF report

## Figures and Tables

**Table 1:** Hydrogen-bond geometry (Å, °) *Cg*2 is the centroid of the C1–C6 benzene ring.

*D*—H⋯*A*	*D*—H	H⋯*A*	*D*⋯*A*	*D*—H⋯*A*
C4—H4⋯O3^i^	0.974 (16)	2.526 (16)	3.4713 (17)	163.6 (11)
C5—H5⋯O3	0.992 (15)	2.592 (16)	3.5435 (15)	160.7 (12)
C14—H14⋯O1	0.963 (15)	2.232 (16)	2.8387 (16)	120.0 (12)
C16—H16*B*⋯O3^ii^	0.993 (14)	2.553 (14)	3.3632 (16)	138.7 (10)
C18—H18*A*⋯*Cg*2^iii^	0.97 (2)	2.93 (2)	3.7585 (17)	144.2 (17)

Symmetry codes: (i) -x+2, -y+1, -z+1; (ii) x-1, y, z; (iii) -x+1, -y+1, -z+1.

**Table 2:** Experimental details

Crystal data	
Chemical formula	C_18_H_16_N_2_O_3_
*M*_r_	308.33
Crystal system, space group	Triclinic, *P*\overline{1}
Temperature (K)	120
*a*, *b*, *c* (Å)	5.3518 (6), 11.6989 (14), 13.3527 (16)
α, β, γ (°)	64.019 (2), 80.323 (2), 76.952 (2)
*V* (Å^3^)	729.83 (15)
*Z*	2
Radiation type	Mo *K*α
μ (mm^−1^)	0.10
Crystal size (mm)	0.42 × 0.18 × 0.12

Data collection	
Diffractometer	Bruker SMART APEX CCD
Absorption correction	Multi-scan (*SADABS*; Krause *et al.*, 2015 ▸)
*T*_min_, *T*_max_	0.87, 0.99
No. of measured, independent and observed [*I* > 2σ(*I*)] reflections	14193, 3909, 2929
*R*_int_	0.028
(sin θ/λ)_max_ (Å^−1^)	0.688

Refinement	
*R*[*F* ^2^ > 2σ(*F* ^2^)], *wR*(*F* ^2^), *S*	0.049, 0.142, 1.00
No. of reflections	3909
No. of parameters	272
H-atom treatment	All H-atom parameters refined
Δρ_max_, Δρ_min_ (e Å^−3^)	0.47, −0.23

Computer programs: *APEX3* and *SAINT* (Bruker, 2016 ▸), *SHELXT* (Sheldrick, 2015*a* ▸), *SHELXL2018/1* (Sheldrick, 2015*b* ▸), *DIAMOND* (Brandenburg & Putz, 2012 ▸) and *SHELXTL* (Sheldrick, 2008 ▸).

## supplementary crystallographic information

### Crystal data

**Table:**

C_18_H_16_N_2_O_3_	*Z* = 2
*M**_r_* = 308.33	*F*(000) = 324
Triclinic, *P*1	*D*_x_ = 1.403 Mg m^−^^3^
*a* = 5.3518 (6) Å	Mo *K*α radiation, λ = 0.71073 Å
*b* = 11.6989 (14) Å	Cell parameters from 5207 reflections
*c* = 13.3527 (16) Å	θ = 3.1–29.3°
α = 64.019 (2)°	µ = 0.10 mm^−^^1^
β = 80.323 (2)°	*T* = 120 K
γ = 76.952 (2)°	Column, colourless
*V* = 729.83 (15) Å^3^	0.42 × 0.18 × 0.12 mm

### Data collection

**Table:**

Bruker SMART APEX CCD diffractometer	3909 independent reflections
Radiation source: fine-focus sealed tube	2929 reflections with *I* > 2σ(*I*)
Graphite monochromator	*R*_int_ = 0.028
Detector resolution: 8.3333 pixels mm^-1^	θ_max_ = 29.3°, θ_min_ = 1.7°
φ and ω scans	*h* = −7→7
Absorption correction: multi-scan (*SADABS*; Krause *et al.*, 2015)	*k* = −16→16
*T*_min_ = 0.87, *T*_max_ = 0.99	*l* = −18→18
14193 measured reflections	

### Refinement

**Table:**

Refinement on *F*^2^	Primary atom site location: structure-invariant direct methods
Least-squares matrix: full	Secondary atom site location: difference Fourier map
*R*[*F*^2^ > 2σ(*F*^2^)] = 0.049	Hydrogen site location: difference Fourier map
*wR*(*F*^2^) = 0.142	All H-atom parameters refined
*S* = 1.00	*w* = 1/[σ^2^(*F*_o_^2^) + (0.0999*P*)^2^] where *P* = (*F*_o_^2^ + 2*F*_c_^2^)/3
3909 reflections	(Δ/σ)_max_ < 0.001
272 parameters	Δρ_max_ = 0.47 e Å^−^^3^
0 restraints	Δρ_min_ = −0.23 e Å^−^^3^

### Special details

**Table:**

Experimental. The diffraction data were obtained from 3 sets of 400 frames, each of width 0.5° in ω, colllected at φ = 0.00, 90.00 and 180.00° and 2 sets of 800 frames, each of width 0.45° in φ, collected at ω = –30.00 and 210.00°. The scan time was 30 sec/frame.
Geometry. All esds (except the esd in the dihedral angle between two l.s. planes) are estimated using the full covariance matrix. The cell esds are taken into account individually in the estimation of esds in distances, angles and torsion angles; correlations between esds in cell parameters are only used when they are defined by crystal symmetry. An approximate (isotropic) treatment of cell esds is used for estimating esds involving l.s. planes.
Refinement. Refinement of F^2^ against ALL reflections. The weighted R-factor wR and goodness of fit S are based on F^2^, conventional R-factors R are based on F, with F set to zero for negative F^2^. The threshold expression of F^2^ > 2sigma(F^2^) is used only for calculating R-factors(gt) etc. and is not relevant to the choice of reflections for refinement. R-factors based on F^2^ are statistically about twice as large as those based on F, and R- factors based on ALL data will be even larger.

### Fractional atomic coordinates and isotropic or equivalent isotropic displacement parameters (Å^2^)

**Table:**

	*x*	*y*	*z*	*U*_iso_*/*U*_eq_	
O1	0.24048 (17)	0.11347 (8)	0.45539 (7)	0.0287 (2)	
O2	0.42582 (17)	0.24290 (8)	0.68691 (6)	0.0264 (2)	
O3	0.74296 (17)	0.36037 (9)	0.62940 (7)	0.0308 (2)	
N1	0.52749 (18)	0.24890 (9)	0.40271 (7)	0.0209 (2)	
N2	0.46952 (18)	0.31557 (9)	0.18053 (7)	0.0205 (2)	
C1	0.6193 (2)	0.37704 (11)	0.20843 (9)	0.0198 (2)	
C2	0.7488 (2)	0.47088 (11)	0.12307 (9)	0.0229 (3)	
H2	0.731 (3)	0.4919 (13)	0.0454 (12)	0.033 (4)*	
C3	0.9057 (2)	0.53135 (11)	0.14791 (10)	0.0253 (3)	
H3	0.999 (3)	0.5940 (13)	0.0898 (11)	0.025 (3)*	
C4	0.9332 (2)	0.50106 (12)	0.25984 (10)	0.0240 (3)	
H4	1.043 (3)	0.5462 (13)	0.2757 (11)	0.023 (3)*	
C5	0.8070 (2)	0.40967 (11)	0.34551 (10)	0.0229 (3)	
H5	0.835 (3)	0.3898 (15)	0.4234 (13)	0.035 (4)*	
C6	0.6527 (2)	0.34508 (11)	0.32082 (9)	0.0197 (2)	
C7	0.3624 (2)	0.19025 (11)	0.37929 (9)	0.0209 (2)	
C8	0.3496 (2)	0.22703 (11)	0.25807 (9)	0.0193 (2)	
C9	0.2018 (2)	0.16113 (11)	0.22093 (9)	0.0210 (2)	
C10	0.2376 (3)	0.18294 (12)	0.10782 (10)	0.0268 (3)	
H10	0.365 (3)	0.2385 (15)	0.0602 (13)	0.040 (4)*	
C11	0.1007 (3)	0.12852 (13)	0.06609 (10)	0.0307 (3)	
H11	0.131 (3)	0.1475 (15)	−0.0164 (14)	0.045 (4)*	
C12	−0.0746 (3)	0.05042 (12)	0.13563 (11)	0.0299 (3)	
H12	−0.174 (3)	0.0113 (13)	0.1067 (11)	0.026 (3)*	
C13	−0.1069 (2)	0.02574 (12)	0.24773 (11)	0.0282 (3)	
H13	−0.229 (3)	−0.0255 (14)	0.2925 (12)	0.030 (4)*	
C14	0.0284 (2)	0.08023 (11)	0.29077 (10)	0.0239 (3)	
H14	−0.004 (3)	0.0579 (14)	0.3697 (12)	0.030 (4)*	
C15	0.5682 (2)	0.20405 (12)	0.52109 (9)	0.0224 (3)	
H15A	0.751 (3)	0.2084 (12)	0.5233 (10)	0.019 (3)*	
H15B	0.546 (2)	0.1157 (14)	0.5606 (11)	0.023 (3)*	
C16	0.3790 (2)	0.28659 (12)	0.57099 (9)	0.0246 (3)	
H16A	0.388 (3)	0.3773 (14)	0.5287 (11)	0.027 (3)*	
H16B	0.200 (3)	0.2750 (12)	0.5718 (10)	0.024 (3)*	
C17	0.6177 (2)	0.28800 (12)	0.70394 (10)	0.0246 (3)	
C18	0.6508 (3)	0.23944 (15)	0.82550 (11)	0.0319 (3)	
H18A	0.545 (4)	0.305 (2)	0.8477 (19)	0.085 (7)*	
H18B	0.600 (4)	0.1579 (19)	0.8692 (16)	0.063 (5)*	
H18C	0.829 (4)	0.2289 (18)	0.8386 (15)	0.060 (5)*	

### Atomic displacement parameters (Å^2^)

**Table:**

	*U*^11^	*U*^22^	*U*^33^	*U*^12^	*U*^13^	*U*^23^
O1	0.0367 (5)	0.0329 (5)	0.0186 (4)	−0.0178 (4)	0.0010 (3)	−0.0080 (4)
O2	0.0315 (5)	0.0335 (5)	0.0192 (4)	−0.0123 (4)	0.0016 (3)	−0.0136 (4)
O3	0.0309 (5)	0.0397 (5)	0.0282 (4)	−0.0145 (4)	0.0022 (4)	−0.0175 (4)
N1	0.0248 (5)	0.0241 (5)	0.0160 (4)	−0.0082 (4)	0.0008 (4)	−0.0094 (4)
N2	0.0230 (5)	0.0203 (5)	0.0195 (4)	−0.0044 (4)	−0.0001 (4)	−0.0097 (4)
C1	0.0212 (5)	0.0209 (5)	0.0188 (5)	−0.0040 (4)	0.0007 (4)	−0.0102 (4)
C2	0.0267 (6)	0.0224 (6)	0.0191 (5)	−0.0050 (5)	0.0012 (4)	−0.0089 (4)
C3	0.0282 (6)	0.0221 (6)	0.0236 (6)	−0.0081 (5)	0.0039 (5)	−0.0080 (5)
C4	0.0239 (6)	0.0247 (6)	0.0280 (6)	−0.0063 (5)	−0.0005 (4)	−0.0147 (5)
C5	0.0241 (6)	0.0256 (6)	0.0220 (5)	−0.0051 (4)	−0.0004 (4)	−0.0127 (5)
C6	0.0203 (5)	0.0205 (5)	0.0185 (5)	−0.0043 (4)	0.0017 (4)	−0.0091 (4)
C7	0.0237 (6)	0.0216 (5)	0.0191 (5)	−0.0063 (4)	0.0002 (4)	−0.0097 (4)
C8	0.0209 (5)	0.0201 (5)	0.0178 (5)	−0.0029 (4)	−0.0013 (4)	−0.0092 (4)
C9	0.0225 (6)	0.0200 (5)	0.0217 (5)	−0.0020 (4)	−0.0038 (4)	−0.0097 (4)
C10	0.0338 (7)	0.0276 (6)	0.0223 (5)	−0.0105 (5)	−0.0018 (5)	−0.0110 (5)
C11	0.0415 (7)	0.0316 (7)	0.0246 (6)	−0.0096 (5)	−0.0061 (5)	−0.0141 (5)
C12	0.0322 (7)	0.0285 (6)	0.0373 (7)	−0.0059 (5)	−0.0088 (5)	−0.0188 (6)
C13	0.0266 (6)	0.0269 (6)	0.0337 (6)	−0.0085 (5)	−0.0009 (5)	−0.0137 (5)
C14	0.0235 (6)	0.0243 (6)	0.0246 (6)	−0.0049 (4)	−0.0009 (4)	−0.0109 (5)
C15	0.0265 (6)	0.0247 (6)	0.0170 (5)	−0.0068 (5)	−0.0007 (4)	−0.0089 (4)
C16	0.0254 (6)	0.0309 (6)	0.0202 (5)	−0.0069 (5)	−0.0011 (4)	−0.0122 (5)
C17	0.0245 (6)	0.0294 (6)	0.0241 (5)	−0.0037 (5)	−0.0003 (4)	−0.0161 (5)
C18	0.0388 (8)	0.0373 (8)	0.0229 (6)	−0.0034 (6)	−0.0053 (5)	−0.0159 (6)

### Geometric parameters (Å, º)

**Table:**

O1—C7	1.2275 (13)	C9—C14	1.3995 (16)
O2—C17	1.3467 (14)	C9—C10	1.4036 (15)
O2—C16	1.4489 (13)	C10—C11	1.3828 (17)
O3—C17	1.2043 (14)	C10—H10	0.990 (16)
N1—C7	1.3800 (14)	C11—C12	1.3884 (19)
N1—C6	1.3882 (14)	C11—H11	1.016 (16)
N1—C15	1.4692 (13)	C12—C13	1.3822 (18)
N2—C8	1.3016 (14)	C12—H12	0.988 (14)
N2—C1	1.3763 (14)	C13—C14	1.3894 (17)
C1—C2	1.4022 (15)	C13—H13	0.934 (15)
C1—C6	1.4114 (14)	C14—H14	0.962 (14)
C2—C3	1.3721 (17)	C15—C16	1.5155 (17)
C2—H2	0.972 (15)	C15—H15A	0.996 (13)
C3—C4	1.4027 (16)	C15—H15B	0.958 (14)
C3—H3	0.962 (13)	C16—H16A	0.967 (14)
C4—C5	1.3795 (16)	C16—H16B	0.993 (14)
C4—H4	0.973 (14)	C17—C18	1.4940 (16)
C5—C6	1.3975 (16)	C18—H18A	0.97 (2)
C5—H5	0.992 (15)	C18—H18B	0.95 (2)
C7—C8	1.4911 (14)	C18—H18C	0.97 (2)
C8—C9	1.4861 (15)		
			
C17—O2—C16	115.33 (9)	C9—C10—H10	117.0 (9)
C7—N1—C6	123.19 (9)	C10—C11—C12	120.53 (12)
C7—N1—C15	116.52 (9)	C10—C11—H11	118.3 (9)
C6—N1—C15	120.28 (9)	C12—C11—H11	121.2 (9)
C8—N2—C1	120.49 (9)	C13—C12—C11	119.10 (11)
N2—C1—C2	119.20 (9)	C13—C12—H12	119.6 (8)
N2—C1—C6	121.67 (10)	C11—C12—H12	121.3 (8)
C2—C1—C6	119.10 (10)	C12—C13—C14	121.02 (12)
C3—C2—C1	120.73 (10)	C12—C13—H13	117.5 (9)
C3—C2—H2	119.6 (8)	C14—C13—H13	121.4 (9)
C1—C2—H2	119.7 (8)	C13—C14—C9	120.32 (11)
C2—C3—C4	119.78 (11)	C13—C14—H14	116.0 (9)
C2—C3—H3	121.2 (8)	C9—C14—H14	123.7 (9)
C4—C3—H3	119.0 (8)	N1—C15—C16	110.08 (9)
C5—C4—C3	120.72 (11)	N1—C15—H15A	106.2 (7)
C5—C4—H4	120.7 (8)	C16—C15—H15A	112.9 (7)
C3—C4—H4	118.5 (8)	N1—C15—H15B	109.3 (8)
C4—C5—C6	119.78 (10)	C16—C15—H15B	110.1 (8)
C4—C5—H5	118.0 (9)	H15A—C15—H15B	108.2 (11)
C6—C5—H5	122.2 (9)	O2—C16—C15	109.30 (10)
N1—C6—C5	122.87 (9)	O2—C16—H16A	111.5 (8)
N1—C6—C1	117.29 (10)	C15—C16—H16A	111.6 (8)
C5—C6—C1	119.84 (10)	O2—C16—H16B	105.9 (7)
O1—C7—N1	120.36 (10)	C15—C16—H16B	110.3 (7)
O1—C7—C8	124.70 (10)	H16A—C16—H16B	108.0 (11)
N1—C7—C8	114.94 (9)	O3—C17—O2	123.41 (10)
N2—C8—C9	117.09 (9)	O3—C17—C18	124.81 (11)
N2—C8—C7	122.12 (10)	O2—C17—C18	111.77 (10)
C9—C8—C7	120.78 (9)	C17—C18—H18A	104.9 (13)
C14—C9—C10	118.14 (10)	C17—C18—H18B	113.1 (11)
C14—C9—C8	124.43 (10)	H18A—C18—H18B	110.9 (18)
C10—C9—C8	117.43 (10)	C17—C18—H18C	111.5 (11)
C11—C10—C9	120.87 (11)	H18A—C18—H18C	110.2 (17)
C11—C10—H10	122.2 (9)	H18B—C18—H18C	106.3 (16)
			
C8—N2—C1—C2	179.20 (10)	O1—C7—C8—N2	175.20 (11)
C8—N2—C1—C6	1.29 (17)	N1—C7—C8—N2	−5.32 (16)
N2—C1—C2—C3	−178.09 (10)	O1—C7—C8—C9	−6.07 (18)
C6—C1—C2—C3	−0.13 (17)	N1—C7—C8—C9	173.42 (9)
C1—C2—C3—C4	−1.29 (18)	N2—C8—C9—C14	−168.77 (10)
C2—C3—C4—C5	0.91 (18)	C7—C8—C9—C14	12.44 (17)
C3—C4—C5—C6	0.92 (18)	N2—C8—C9—C10	10.61 (16)
C7—N1—C6—C5	175.87 (10)	C7—C8—C9—C10	−168.19 (10)
C15—N1—C6—C5	−4.42 (16)	C14—C9—C10—C11	1.58 (18)
C7—N1—C6—C1	−4.13 (16)	C8—C9—C10—C11	−177.84 (11)
C15—N1—C6—C1	175.58 (10)	C9—C10—C11—C12	−0.4 (2)
C4—C5—C6—N1	177.66 (10)	C10—C11—C12—C13	−1.2 (2)
C4—C5—C6—C1	−2.34 (17)	C11—C12—C13—C14	1.52 (19)
N2—C1—C6—N1	−0.14 (16)	C12—C13—C14—C9	−0.28 (19)
C2—C1—C6—N1	−178.05 (10)	C10—C9—C14—C13	−1.26 (17)
N2—C1—C6—C5	179.86 (10)	C8—C9—C14—C13	178.11 (10)
C2—C1—C6—C5	1.95 (16)	C7—N1—C15—C16	−92.28 (12)
C6—N1—C7—O1	−173.92 (10)	C6—N1—C15—C16	88.00 (12)
C15—N1—C7—O1	6.36 (16)	C17—O2—C16—C15	82.16 (12)
C6—N1—C7—C8	6.57 (15)	N1—C15—C16—O2	−178.70 (9)
C15—N1—C7—C8	−173.15 (9)	C16—O2—C17—O3	0.52 (16)
C1—N2—C8—C9	−177.24 (9)	C16—O2—C17—C18	179.37 (10)
C1—N2—C8—C7	1.54 (17)		

### Hydrogen-bond geometry (Å, º)

*Cg*2 is the centroid of the C1–C6 benzene ring.

**Table:**

*D*—H···*A*	*D*—H	H···*A*	*D*···*A*	*D*—H···*A*
C4—H4···O3^i^	0.974 (16)	2.526 (16)	3.4713 (17)	163.6 (11)
C5—H5···O3	0.992 (15)	2.592 (16)	3.5435 (15)	160.7 (12)
C14—H14···O1	0.963 (15)	2.232 (16)	2.8387 (16)	120.0 (12)
C16—H16*B*···O3^ii^	0.993 (14)	2.553 (14)	3.3632 (16)	138.7 (10)
C18—H18*A*···*Cg*2^iii^	0.97 (2)	2.93 (2)	3.7585 (17)	144.2 (17)

Symmetry codes: (i) −*x*+2, −*y*+1, −*z*+1; (ii) *x*−1, *y*, *z*; (iii) −*x*+1, −*y*+1, −*z*+1.
